# Evidence-base for urban green-blue infrastructure to support insect diversity

**DOI:** 10.1007/s11252-024-01649-4

**Published:** 2024-12-07

**Authors:** Diana E. Bowler, Corey T. Callaghan, Jéssica F. Felappi, Brittany M. Mason, Robin Hutchinson, Prashant Kumar, Laurence Jones

**Affiliations:** 1 Biodiversity Monitoring & Analysis, UK Centre for Ecology & Hydrology, Wallingford, UK; 2 Department of Wildlife Ecology and Conservation, Fort Lauderdale Research and Education Center, University of Florida, Gainesville, FL USA; 3 Center for Development Research, University of Bonn, Bonn, Germany; 4 Department of Civil and Environmental Engineering, Faculty of Engineering and Physical Sciences, Global Centre for Clean Air Research (GCARE), University of Surrey, Guildford, GU2 7XH UK; 5 Institute for Sustainability, University of Surrey, Guildford, GU2 7XH UK; 6 UK Centre for Ecology & Hydrology, Environment Centre Wales, Bangor, LL57 2UW UK; 7 Department of Geography and Environmental Science, Liverpool Hope University, Hope Park, Liverpool, L16 9JD UK

**Keywords:** Blue-green infrastructure, Gardens, Invertebrates, Nature-based solutions, Parks, Urban biodiversity, Urban green space, Urbanisation, Urban planning

## Abstract

Green-blue urban infrastructures potentially offer win-win benefits for people and nature in urban areas. Given increasing evidence of widespread declines of insects, as well as their ecological importance, there is a need to better understand the potential role of green-blue urban infrastructure for insect conservation. In this review, we evaluated 201 studies about the ability of green-blue infrastructure to support insect diversity. Most studies were focused on the role of local and landscape-level characteristics of green-blue infrastructure. Fewer studies explicitly compared one type of infrastructure to another, and even fewer compared insect communities between green-blue infrastructure and traditional infrastructure. Overall, the body of research highlights the importance of plant diversity and reduced intensity of management (e.g., mowing) for most insect taxon groups. While local characteristics seem to be generally more important than landscape factors, insect communities within green-blue infrastructures can also depend on their connectivity and landscape context. Some infrastructure types are generally more beneficial than others; for instance, ground-level habitats tend to support more insects than green roofs. Few studies simultaneously studied synergies or trade-offs with other services provided by green-blue infrastructure, but environmental variables, such as tree cover and plant diversity, that affect insects are likely to also affect the provision of other services such as improving thermal comfort and the well-being of people. Our review offers some initial evidence for how green-blue infrastructure could be designed for multifunctionality with insects in mind.

**Supplementary Information:**

The online version contains supplementary material available at 10.1007/s11252-024-01649-4.

## Introduction

Evidence has accumulated for widespread declines of insect populations and communities (van Klink et al. 2020). The estimated magnitude of the declines has raised concern from scientists, policymakers and the public alike because of the possible implications for ecosystem services, such as pollination (Cardoso et al. 2020), and cascading effects on species reliant on insects, such as many bird species (Grames et al. 2023). A diverse range of threats to insects have been identified, with changes in land-use typically assessed as the most pressing threat (Wagner et al. 2021). Disentangling the impacts of different drivers of declines is, however, challenging for a range of reasons, including the lack of large-scale insect monitoring and their typically large inter-annual fluctuations. Nonetheless, there are already calls for immediate action to reverse declines (Forister et al. 2019; Kawahara et al. 2021). To inform this action, there is a need to consolidate the evidence-base for interventions that promote insect diversity.

Urbanisation is a widespread threat to many insect populations (Fenoglio et al. 2020; Svenningsen et al. 2022; Vaz et al. 2023). Urbanisation tends to negatively affect multiple facets of insect diversity, including total insect biomass (Uhler et al. 2021; Svenningsen et al. 2022), and species richness and diversity (Fenoglio et al. 2020). Multiple mechanisms are at play, including changes in vegetation cover, habitat fragmentation, and increased pollutants (Fenoglio et al. 2021; Collins et al. 2024). Compared to other land covers, urban cover tends to be a relatively small fraction of the land area in most countries, which means that urbanisation alone is not likely responsible for many insect declines. However, because urbanisation is increasing at a rapid rate (Sun et al. 2020), it is critical to consider how the negative impacts of urbanization can be reduced and to develop urban biodiversity conservation strategies.

Urban green-blue infrastructure, composed of different types of small or large semi-natural areas, is an important component of urban planning, offering potentially diverse benefits for both people and nature (Pauleit et al. 2020). Urban green-blue infrastructure is also recognised as a nature-based solution for climate change adaptation, tackling the impacts of heatwaves, as well as biodiversity loss (Castellar et al. 2021; Goodwin et al. 2023). Such infrastructures include multi-use areas such as gardens and parks, recreational and food production areas, as well as small green spaces such as green roofs, road verges and street trees (Castellar et al. 2021; Jones et al. 2022). As recognition of their value, such infrastructure are included in international targets for sustainable cities and biodiversity (e.g., Target 12 in the Kunming-Montreal Global Biodiversity Framework). Also at local-scales, multiple toolkits have been developed to facilitate decision-making and planning (Van Oijstaeijen et al. 2020; Derickson et al. 2021), although planning documents often lack measurable targets (Nilon et al. 2017) in part because of a lack of a quantifiable understanding of how infrastructure influences different components of biodiversity.

While there have been some reviews of the benefits to biodiversity of some urban green-blue infrastructure (Beninde et al. 2015; Filazzola et al. 2019), few have focused on insects; surprising given the noted declines of insects globally. Moreover, many reviews have focused on only a subset of possible green-blue infrastructure types. Here, we aimed to fill this gap by mapping the evidence-base for a diverse range of urban green-blue infrastructures to support insect diversity. Our purpose was to provide a qualitative overview that summarises the current approaches and methodologies, while highlighting key take-aways from the current literature. We also aimed to assess the evidence for co-benefits of green-blue infrastructure for insect diversity and other possible services to people.

## Methods

To structure our literature search, we independently searched for studies by different infrastructure types. We used a recently developed typology (Jones et al. 2022) that covers 49 green-blue infrastructures, grouped into nine broad categories (gardens; parks; amenity areas such as golf courses; other public spaces such as cemeteries and allotments; linear features/routes such as road verges and street trees; constructed infrastructure such as green roofs and walls; hybrid infrastructure such as rain gardens and attenuation ponds; water bodies and other non-sealed urban areas). For each of the 49 types, we searched for relevant articles in Web of Science using the following search string: (urban OR city OR cities OR town*) AND (insect* OR invertebrate* OR arthropod*) AND (richness OR abundance OR biomass) AND infrastructure type (where infrastructure type was replaced with the name of one of the 49 types and any synonyms). We did not add any additional search terms for possible co-benefits or trade-offs since we were only interested in studies that – at a minimum – at least measured an insect outcome. The main searches were performed in February 2023. Studies captured by each search were maintained in separate Endnote reference libraries.

We filtered the studies according to whether they met a set of pre-defined inclusion criteria. These criteria were composed of a relevant outcome (the study had to measure insect diversity in terms of either abundance, richness or biomass); relevant population (the study had to measure insect diversity within an urban area - including peri-urban); relevant intervention (the study had to measure insect diversity within any sort of green or blue infrastructure) and relevant comparator (the study had to compare insect diversity within at least two types of places - this was kept intentionally broad so that we could assess the typical comparisons/questions of the studies). We also defined several specific exclusion criteria: we excluded studies focusing on only one insect species as well as studies focusing on an urban versus rural comparison (or a continuous version e.g., % urban cover). We first applied the inclusion/exclusion criteria, as far as possible, by reading titles/abstracts of the studies in each independent Endnote library. Next, the libraries were pooled, duplicates removed, and the final inclusion/exclusion was assessed on reading the full text. We moved reviews/syntheses that were relevant to the topic but did not necessarily meet all the inclusion criteria into a separate library.

From each included study, we extracted information on city and country of data collection; green-blue infrastructure type (following Jones et al. 2022); focal taxon group; study design and comparison; time period and number of study sites; and main local and/or landscape characteristics investigated (when appropriate). For the latter, we also extracted qualitative information on the study findings on the effects of size/area, isolation/connectivity, plant cover and diversity, and site management. Using the extracted information on the study design/comparison, we clustered the studies into three main groups, primarily distinguished by how sampling sites were selected since that determined the inferences drawn. These three groups were: studies investigating local/landscape characteristics; studies comparing different types of infrastructure; and studies comparing green-blue infrastructure with traditional or grey infrastructure.

Because of the diversity of the studies and the intentional broadness of our goal, we refrained from a quantitative synthesis to avoid ‘apples and oranges’ criticisms and statistical issues in combining data that had been summarised in different ways. Instead, we present a narrative review of the main groups of studies and their findings. When there are many studies asking a particular question, we focus on available meta-analyses or primary research articles comparing the largest number of green spaces. When multiple insect metrics were analysed, we focus on total abundance and richness.

## Results

### Relevant empirical studies

Of 3,519 screened articles, we found 18 relevant reviews / meta-analyses (Table S1) and 183 empirical studies that passed our inclusion criteria. Studies were usually excluded because they focused on the negative effects of urbanisation; for instance, by comparing urban habitat with rural habitat, rather than on possible positive effects of green-blue urban infrastructure.

Most of the studies were conducted within Europe (34%) and North America (32%); the remainder were primarily from Australia and New Zealand (13%) and Asia (7%). Most studies (44%) collected data on multiple insect groups or the whole insect assemblages that were captured by their sampling methods. The rest focused on a diverse range of specific taxonomic groups, including ants, beetles, wasps among many others. Because of the large-scale of most of the green-blue infrastructures of interest, most studies used an observational study design (83%) rather than an experimental study design (17%), for instance, the research was focused on existing infrastructure rather than involving any specific manipulation performed by the authors. See https://diana-bowler.shinyapps.io/Urban_greening_for_insects/ for an interactive data table with full details on the studies.

### Focal green-blue infrastructure

**Fig. 1:**
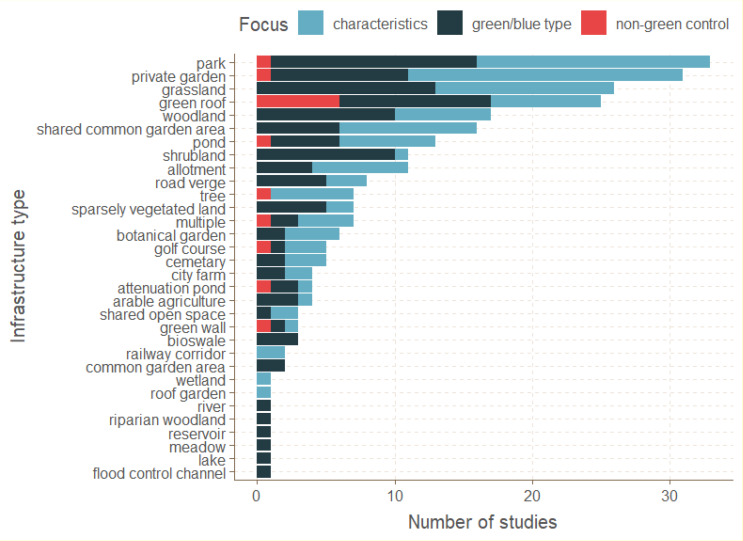
Number of studies that passed our inclusion criteria on each green-blue infrastructure type. Colors refers to different study designs: characteristics = studies testing the effect of different infrastructure characteristics (local and/or landscape characteristics, e.g., different parks that varied in their attributes); green/blue type = studies that compared different types of infrastructures (e.g., parks versus gardens); non-green control = studies that compare green infrastructure with urban non-green. When a study could be placed in multiple categories, we placed it in the category that best matched the primary goal of the study

Most studies targeted managed forms of green-blue infrastructure such as parks or gardens (Fig. 1). Within constructed infrastructure types, green roofs were the most studied. A relatively small number of studies looked at the value of small green spaces such as balconies, or potentially biodiverse green spaces such as cemeteries or botanical gardens (Fig. 1). Overall, green infrastructures were much more studied than blue infrastructures (90% green vs. 10% blue, mostly ponds, Fig. 1).

### Study design and research questions

#### Effects of local and landscape characteristics of green-blue spaces on insect diversity

**Fig. 2:**
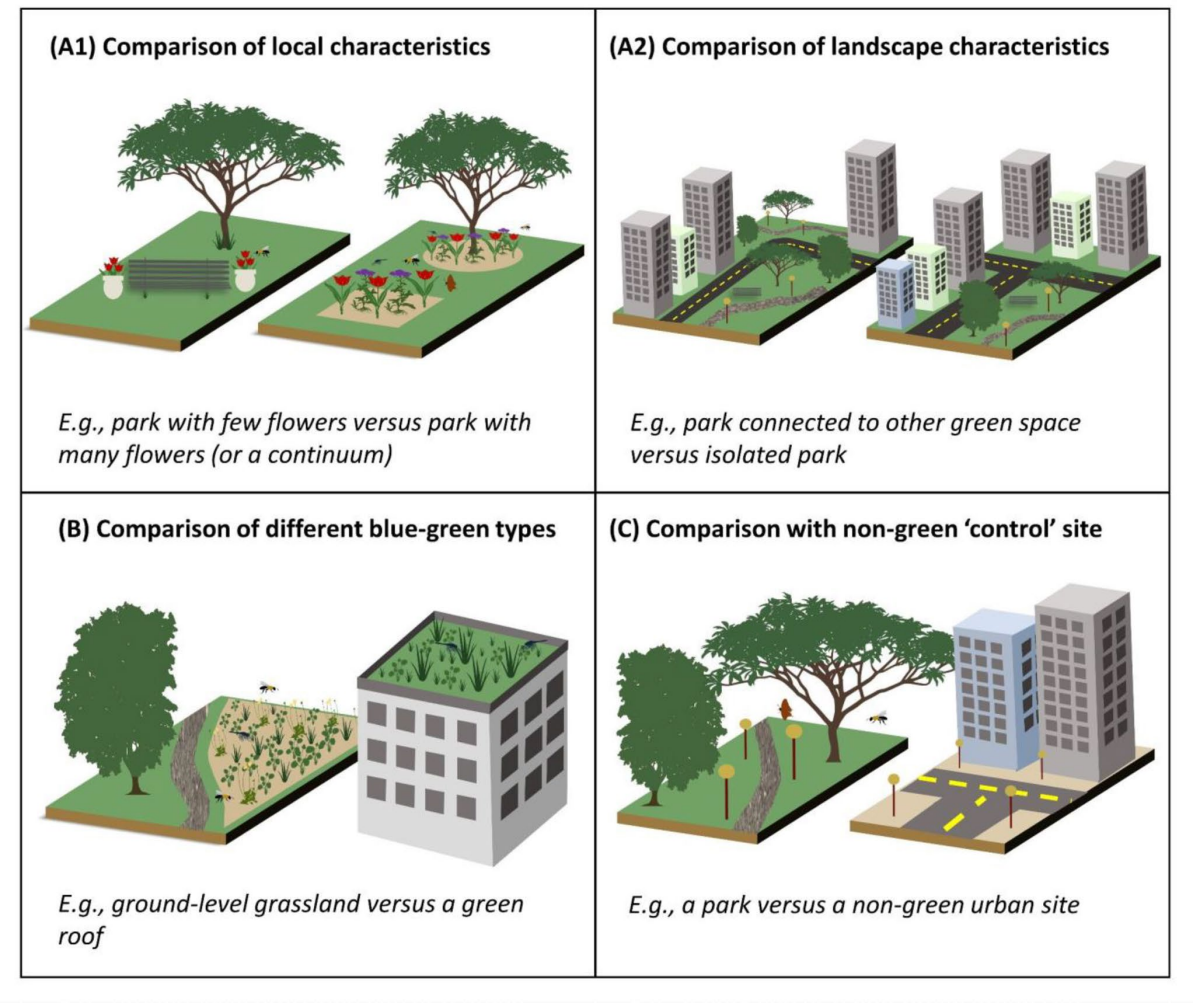
The main types of study designs exploring the ability of urban green-blue infrastructure to support insects: **A1** and **A2** represent studies that tested for the effects of local and landscape characteristics, respectively; **B** represents studies that compared different types of green-blue infrastructure; and **C** represents studies that compared green-blue infrastructure with a primarily non-green control

Most studies fell into this category (58%, Fig. 1). Typically these studies sampled insects in multiple green-blue spaces, usually of the same broad infrastructure type (e.g., parks or gardens), and related the characteristics of these spaces to the local insect community. The characteristics that were tested included both local characteristics, such as area or vegetation cover (Fig. 2A1) as well as landscape-level characteristics, such as land cover in the surrounding region or connectivity with other green spaces (Fig. 2A2). Local characteristics also included specific management practices e.g., mowing regime, or differences between the importance of native versus non-native vegetation.

#### Comparison of insect diversity among different green-blue infrastructure types

These studies (35%, Fig. 1) compared insects across sampling sites within fundamentally different forms of green-blue infrastructure (Fig. 2B). For instance, a group of studies compared insect diversity within or above a green roof with insect diversity within or above a nearby ground-level green site. Another group of studies compared a managed green-blue infrastructure with “vacant”, unmanaged or abandoned land or with remnant natural habitat. Beyond these, there were a range of other comparisons of different infrastructure types, including meadows versus lawns; gardens versus parks; and parks versus woodland.

#### Comparison of insect diversity between green-blue infrastructure and urban built non-green control

These studies (7%) sampled insects within green-blue infrastructure and a control urban site that was primarily non-green. Most of these studies compared green roofs or walls with other types of non-green roofs and walls. For all the other green-blue infrastructure types, few studies compared insect diversity with that in the urban built environment or with conventional infrastructure (Fig. 2C).

### Key findings

#### Value of ground-level infrastructure

Green roofs typically attract more species than a conventional or non-green roof (Partridge and Clark 2018; Schindler et al. 2018; Wooster et al. 2022), a finding also emerging from a focused systematic review (Wang et al. 2022). However, green roofs do not typically support the same amount of insect diversity as ground-level habitat (MacIvor and Lundholm 2011; Quispe and Fenoglio 2015; Braaker et al. 2017; Wong and Jim 2018; Sanchez Dominguez et al. 2020; Gonsalves et al. 2022; Kyro et al. 2022). Consistent with this, studies have reported negative effects of green roof height on insects (Dromgold et al. 2020). But green walls can be as effective as ground-level habitat (Treder et al. 2024).

When comparing among different types of ground-level infrastructure, allotments and gardens typically support more diverse insect communities than parks (Andersson et al. 2007; Lowe et al. 2018; Trigos-Peral et al. 2020). Insect biomass and diversity, however, is still often higher within remnant habitat than in managed green-blue infrastructures (Threlfall et al. 2012; Soga et al. 2014; Lowe et al. 2018; Toft et al. 2019; Shrestha et al. 2021).

Several studies highlight the importance of water bodies, such as ponds (Oertli and Parris 2019; Straka et al. 2020), including stormwater ponds (Hassall and Anderson 2015; Greenway 2017; Holtmann et al. 2018, 2019). However, various factors affect their value for insect biodiversity including pond depth (Heino et al. 2017); size (Hill et al. 2015; Blicharska et al. 2016); diversity of vegetation (Goertzen and Suhling 2013; Thornhill et al. 2017; Chen et al. 2020) and vegetation coverage (Blicharska et al. 2016; Kietzka et al. 2021). Temporary waterbodies are important for some taxa (Fontanarrosa et al. 2009; Holtmann et al. 2019).

#### Importance of site management

Different types of studies indicate that more intensely managed green-blue infrastructures support fewer insects. Management practices can comprise diverse actions including mowing and the use of pesticides and fertilizers (Blubaugh et al. 2011; Huang et al. 2020). Most studies (9 out ot 14) testing the effect of mowing indicated that more intensively mowed lawns or roadside vegetation, typically had lower abundances of insects e.g., (Smith et al. 2015; Moron et al. 2017; Buchholz et al. 2018; Mody et al. 2020; Lange-Kabitz et al. 2021; Wintergerst et al. 2021), which may explain differences between different infrastructure types (Bennett and Lovell 2014; Francoeur et al. 2021). Furthermore, a meta-analysis of 28 studies found that reduced mowing was associated with greater abundance, and especially greater richness, of arthropod communities (Proske et al. 2022). Also, vacant land or lots, which are minimally managed/mown, typically have higher insect diversity and abundance than similar nearby more managed green spaces (Robinson and Lundholm 2012; Riley et al. 2018). Other studies highlight the importance of allowing some ‘spontaneous vegetation’, i.e., plant species independently colonizing, for local insect communities (Nagase et al. 2018). Other studies highlight the importance of considering mowing time (Horstmann et al. 2024) and allowing some occasional mowing or grazing, e.g., by mammalian herbivores, for promoting diversity in grassland habitats. For instance, managed (mowed) urban orchards had higher diversity across multiple insect taxa than abandoned ones (Rada et al. 2023).

Management decisions can also involve choices around seed or plant additions (Turo and Gardiner 2021), and whether and how to manage alien/non-native species. Alien species are common within urban areas, partly because of horticulture and partly because of common invasion pathways. However, there is a diversity of findings regarding the importance of native versus non-native vegetation for insects. Green roofs planted with native grassland did not differ from those planted with non-native species (Dromgold et al. 2020). But a few studies did report negative effects of non-native vegetation on insect diversity (Smith et al. 2015; Jensen et al. 2022), and indicate that native vegetation is important in some contexts (Helden et al. 2012; Mata et al. 2021; Turo and Gardiner 2021). A meta-analysis corroborated this tendency towards negative impacts of non-native species for urban bees (Prendergast et al. 2022). However, another meta-analysis found mixed impacts of alien plant species on insect pollinators (Majewska and Altizer 2020). Overall, several studies indicate that plant resources are more important than plant origin, at least from the insect perspective (Matteson and Langellotto 2011; Berthon et al. 2021). More generally insect richness and/or abundance is positively associated with total plant richness (Table 1), see also (Braaker et al. 2017; Lanner et al. 2020).

**Table 1:** Functional insect groups examined by the studies

Ecosystem service/ disservice	Key associated insect taxa	Positively affected by …	Key References
Pollination	Bees, Hoverflies, Butterflies (to a lesser extent)	Flowering plant cover and diversity; Plant richness; Habitat size	(Shwartz et al. 2013; Buchholz et al. 2020; Daniels et al. 2020; Lanner et al. 2020; Majewska and Altizer 2020; McCune et al. 2020; Lange-Kabitz et al. 2021; Turo and Gardiner 2021; Griffiths-Lee et al. 2022; Prendergast et al. 2022; Watson et al. 2022; Horstmann et al. 2024)
Biological pest control	Wasps, Lady birds, Various parasitoids	Plant cover and diversity; Flowering plant cover; Vegetation complexity; Habitat size	(Bennett and Lovell 2014; Burks and Philpott 2017; Mata et al. 2017; Lowenstein and Minor 2018; Rocha et al. 2018; Wan et al. 2018; Frank et al. 2019; Dale et al. 2020; Philpott et al. 2020; Nighswander et al. 2021; Arnold 2022; Egerer and Philpott 2022)
Disease vectors	Mosquitoes, ticks	Green space area; Ground-level habitat	(Wong and Jim 2016, 2018; Medeiros-Sousa et al. 2017; Yang et al. 2019)
Decomposition	Carrion flies, carrion beetles, rove beetles		None found.

#### Green-blue infrastructure as islands

Many of the studies on local and landscape characteristics tested for the effects of area and isolation of green-blue infrastructure. Area and isolation are predicted to be important based on the classic ecological theory of island biogeography, originally developed for oceanic islands (MacArthur and Wilson 1967), but adapted to the urban context by considering green-blue infrastructures as islands within the urban matrix (Blank et al. 2017; Fattorini et al. 2018). Based on this theory, area is predicted to be positively associated with the number of species, while isolation is predicted to be negatively associated.

Many, but not all, studies were consistent with these predictions. Land area of the green-blue infrastructure was found to be positively associated with insect abundance or richness in 19 studies (see Table S1), including studies comparing a large number of sites [e.g., 214 gardens in the UK, (Bates et al. 2014); 115 green roofs across France, (Madre et al. 2013)]. However, 24 studies found mixed evidence, with positive association between area and insects for only some of the sampled taxa [e.g., across 80 green roofs/ground-level habitats, (Braaker et al. 2017)]; or there was only a weak effect [e.g. within roadside vegetation, (Mody et al. 2020)]. Nufio et al. (2009) et al. also showed that smaller urban habitat fragments had fewer grasshopper species, controlling for the number of sampled individuals. Similarly, isolation of a green-blue infrastructure - in terms of its distance from other green-blue infrastructure - was negatively associated with insect abundance or richness in 7 studies [e.g., comparing 8 green roofs/ground-level habitats in Zurich, (Braaker et al. 2017), and 30 urban grasslands in Berlin, (Buchholz et al. 2020)], but it had a mixed or weak importance in another 12 other studies [e.g., across 46 green spaces in Perth, (Williams 2011)].

#### Landscape context matters, but less so than local factors

Most of the studies exploring the effect of local characteristics simultaneously tested the effects of one or more landscape-level characteristics, including the land cover in the surroundings. These studies indicate that the same green-blue infrastructure type can support different insect communities depending on where it is placed in the urban landscape. For instance, 13 studies found that greater urban cover surrounding a green-blue infrastructure led to lower species richness or abundance within it [e.g., across 214 gardens in the UK, (Bates et al. 2014)]; see also (Hostetler et al. 2011; Lagucki et al. 2017; Philpott et al. 2019; Braschler et al. 2020; Biella et al. 2022; Horstmann et al. 2024; Kaiser and Resasco 2024). However, 10 studies did not find any effect [e.g., across 96 sites across Swiss cities, (Sattler et al. 2010)]. Overall, studies comparing local and landscape features found that local factors (e.g., vegetation cover and site management) were generally more important than landscape factors (Strauss and Biedermann 2006; Shwartz et al. 2013; Lintott et al. 2014; Philpott et al. 2014; Otoshi et al. 2015; Kyro et al. 2018; Lanner et al. 2020; Lin and Chen 2022; Watson et al. 2022; Huchler et al. 2023). Indeed, two meta-analyses agreed that local factors were most important; for instance, for pollinators in gardens (Majewska and Altizer 2020) and more generally across green urban areas for biodiversity (Beninde et al. 2015).

#### Variation among taxa and implications for ecosystem services

The importance of local characteristics, such as flowering plant diversity and tree cover, vary among species, taxon groups and functional groups (McIntyre et al. 2001; Smith et al. 2006a, b; Mata et al. 2017; Braschler et al. 2020; Trigos-Peral et al. 2020). Some studies highlight the needs of specialist species, for instance, regarding specific host plant and habitat requirements (Robinson and Lundholm 2012; Turrini and Knop 2015; Rada et al. 2023), e.g., large wooded areas for saproxylic beetles (Fattorini et al. 2018). At the same time, some studies did identify common responses across different insect groups. For instance, multiple groups are positively affected by flower or plant species richness (Robinson and Lundholm 2012; Tamara et al. 2021) and by increasing habitat area (Sanchez Dominguez et al. 2020).

Studies targeting specific functional groups have the potential to reveal factors affecting the provision of specific ecosystem services (Table 1). The most common target functional group was pollinators, including bees, hoverflies and butterflies (22%), which were especially well-studied within parks and gardens. A smaller group of studies (9%) focused on natural enemies (a range of parasitoids / predators from different taxonomic groups) and their ability to provide biological pest control services of herbivorous insects, usually within allotments or urban farms. Similar variables, including plant diversity and cover, promote both pollinators and natural enemies (Table 1). We also found studies linking green space design to mosquito abundance (Table 1). None of the included studies investigated decomposition services of insects within green-blue infrastructure. We note that because we did not perform taxon-specific literatures searches, but searched for studies more generally on insects, our coverage is likely to underestimate the literature available for specific insect groups.

Based on the diverse needs of insects, several studies highlighted the importance of habitat heterogeneity within infrastructures, offering diverse microclimates and habitats, to support a diverse insect community (Bates et al. 2014; Leonard et al. 2018; Abbate et al. 2019). This might explain the high insect diversity found in green-blue infrastructure with frequent high diversity of vegetation structure and plant species composition, such as gardens (Daniels et al. 2020; Trigos-Peral et al. 2020).

#### Synergies and trade-offs with other ecosystem services

Only a few studies considered other ecosystem services or benefits of green-blue infrastructure along with insect diversity (Table 2). The exceptions considered heat wave mitigation, stormwater management and cultural or recreational services for people. Francoeur et al. (2021) compared the ability of different green infrastructure to support biodiversity and provide heat mitigation. They found that flower meadows, instead of lawns, were beneficial to both arthropods, shown as higher biomass and richness, as well as heat mitigation, shown by lower surface temperatures. Highly maintained lawns, by contrast, had both higher temperatures and lower arthropod biomass (Francoeur et al. 2021). Infrastructure designed for stormwater/flooding management can also support insect diversity. For instance, Kazemi et al. (2009) and (2011) found street-side bioretention basins/swales supported more insects than nearby lawns, which was explained by greater habitat heterogeneity. Ge et al. (2019) found that biofilters were usually intermediate, in the insect communities that they could support, between lawns and natural habitats. In other cases, constructed infrastructures for flood/stormwater management were found to support similar amounts of biodiversity as nearby semi-natural habitat (Hassall and Anderson 2015; Holtmann et al. 2018). For instance, Moron et al. (2017) found similar butterfly diversity on levees and nearby grasslands, both maintained with low intensity management.

**Table 2:** Potential synergies with insect-friendly green-blue infrastructure

Service	Associated infrastructure	References
Heat mitigation	Flower meadows	(Francoeur et al. 2021)
Stormwater management	Bioretention basins; Swales; Biofilters; Levees; Stormwater ponds	(Kazemi et al. 2009, 2011; Hassall and Anderson 2015; Moron et al. 2017; Holtmann et al. 2018; Ge et al. 2019)
Public perception / well-being	Flower meadows	(Shwartz et al. 2014; Garbuzov et al. 2015; Hoyle et al. 2018)

Green-blue infrastructure can also offer cultural and recreation services that promote public health and well-being. We did not find any study that directly tested for insect outcomes and human health and well-being outcomes, but some studies did at least study human perceptions. Hoyle et al. (2018) studied the characteristics of meadows associated with aesthetic preferences of people and with insect diversity. Flower color diversity was positively associated with pollinators and preferred by people; however, flower diversity itself was only important for pollinators. The authors recommended incorporating late-flowering non-native species into seed mixes to benefit both people and insects. Schwartz et al. (2014) suggested that biodiversity *per se* might not be accurately perceived by people, but that appreciation for biodiversity has various nuances, as people preferred gardens rich in flowers, trees, and birds, rather than insects. Finally, Marshall et al. (2023) found meadows, compared to lawns, had higher species richness (including insects), lower greenhouse gas emissions, lower maintenance costs, higher solar reflectance, were more aesthetically pleasing and better for mental well-being, but offered fewer recreational services. We note that since we did not specifically search for studies on co-benefits that we may have missed some studies that have relevance for insects.

## Discussion

### Implications for urban planning and policy

Our review aimed for breadth rather than depth, but our results still allow us to highlight some specific findings with relevance for urban planning. First, our review indicates that green roofs usually support fewer insects than ground-level habitat (Braaker et al. 2017). This difference may partly stem from elevation affecting accessibility, coupled with the small area of many green roofs. While green roofs are an attractive option to retro-fit green space into urban areas, this finding means that they should not be seen as a replacement for insect-friendly ground-level habitat. But green roofs do offer more resources than a traditional roof, so they still have a value for urban biodiversity (Wang et al. 2022). Second, urban grasslands are often managed too intensively to provide suitable habitat and resources for biodiversity, largely explained by mowing and other management practices (e.g., fertilizer and herbicides) (Buchholz et al. 2018). By contrast, we found widespread evidence of the value of wildflower meadows for insects, even in relatively small areas such as roadside verges (Mody et al. 2020; Brown et al. 2024). Third, while landscape context matters, local characteristics of the infrastructure, especially vegetation cover and diversity, appear to dominate in shaping the local insect communities (Majewska and Altizer 2020). This gives some support for opportunistic conversion of available plots throughout the urban matrix into green-blue infrastructure (Turrini and Knop 2015). Finally, meeting the diverse needs of insects requires creating habitat and resource heterogeneity, which will need careful spatial planning at multiple spatial scales. Taken together, our findings illustrate the importance of distinct and focused planning for insects in urban green-blue infrastructure.

### Multifunctionality of green-blue infrastructure

Urban planning usually prioritizes services related to human use, while the emerging One Health concept advocates that the health of humans, animals, and the environment are intrinsically connected (Queenan et al. 2017) and effective sustainable solutions should consider benefits and threats to the three dimensions. Similarly, the definition of nature-based solutions includes an explicit element of supporting biodiversity (Cohen-Shacham et al. 2016; Skodra et al. 2021). The multifunctionality of green-blue infrastructure is embedded in its concept (Tzoulas et al. 2007) and explains why it has become one of the main strategies to improve environmental quality in cities and enhance climate change resilience. However, planners may face difficult decisions; for instance, if there are trade-offs such that increasing biodiversity might decrease the delivery of another ecosystem service. Alternatively, there may be opportunities for synergism, where interventions that increase biodiversity also increase the delivery of ecosystem services and other benefits (Garbuzov et al. 2015; Francoeur et al. 2021; Jones et al. 2022). Many of the environmental factors affecting insects are also likely to affect the provision of other services. Tree cover can affect insect communities and is known to affect the ability of urban green infrastructure to buffer temperature extremes (De Lombaerde et al. 2022) and is associated with higher psychological restoration (Felappi et al. 2024). Wildflower meadows have potentially broad benefits for people and nature (Bretzel et al. 2016), including reduced maintenance costs (Mody et al. 2020).

### Knowledge gaps and future research directions

Our review highlights several knowledge gaps that could be targeted by future research. First, there are still large geographic gaps in the data, which prevents understanding how different contexts (e.g., climate, elevation, biogeography, extent of development) modify the benefits of green-blue infrastructure in specific countries. In particular, tropical regions are underrepresented. Additionally, despite their prevalence, relatively few studies focused on small infrastructures such as roadside verges and street trees. Also, despite the known importance of freshwater systems for biodiversity, blue infrastructures, such as small waterbodies, are much less studied than green infrastructures. But the available literature indicates the potential for ‘quick-wins’ with these infrastructure types for insect communities (Chester and Robson 2013; Turrini and Knop 2015; Mody et al. 2020; Huchler et al. 2023; Brown et al. 2024). Moreover, the collective value of these small infrastructures across an urban area has not been quantified. Given the prevalence of small green spaces, there is a need to better understand their value to decide which green-blue infrastructure types should be priorities for investment. A further knowledge gap is that most studies focused on simple linear effects of infrastructure characteristics, such as size. This means there is little understanding of whether there are threshold effects that reflect minimum needs or standards to ensure the infrastructure is fit-for-purpose (Beninde et al. 2015). Small patches of green infrastructure can still support insects (Brunbjerg et al. 2018; Horstmann et al. 2024), but few studies attempted to identify threshold effects that could define minimum size requirements.

Our review found that the current evidence base often lacks a study design that enables quantification of the total benefits of green-blue infrastructure. A common approach to evaluate the impacts of an intervention in evidence-based conservation is the BACI (Before-After-Control-Impact) design (Christie et al. 2019). BACI is an observational study design that tracks an outcome at intervention sites as well as at control sites without the intervention, before and after the intervention is implemented. However, its application in urban biodiversity research, especially concerning established green-blue infrastructures, has been limited (but see Marshall et al. 2023; Brown et al. 2024). This can be partly explained by the absence of baseline data i.e., data available before the infrastructure was established. Moreover, there is a lack of extensive sampling in gray or traditional infrastructure spaces, largely because it often does not make sense for extensive insect sampling, since areas outside of green-blue spaces provide few resources for insects and only contain ‘temporary’ dispersing individuals. Nonetheless, base-line data and comparators are essential for quantification of total benefits. To address these gaps, future studies should consider incorporating BACI or similar quasi-experimental designs where feasible. Studies could benefit from using ‘positive controls’ – areas that represent conservation targets, such as semi-natural habitats – and ‘negative controls’ – conventional infrastructure – so the difference with the green-blue infrastructure can be quantified (Filazzola et al. 2019). Collecting longitudinal data, including establishing baseline measurements before new green-blue infrastructures are implemented, could also help our ability to discern causal effects. There are many urban green-blue infrastructure projects ongoing, providing opportunities to build in proper study design to help inform ongoing management for insect diversity.

A related challenge of the evidence-base is the lack of causal framework in the statistical analyses of the data. This means that there is often no explicit consideration of how different local or landscape variables might relate to each other, nor how treatment bias (i.e., where green-blue infrastructures are implemented) might affect the results (Ferraro et al. 2019). Instead, most studies used ‘causal salad’ regression (McElreath 2020) (i.e., all variables were included in a multiple regression model) and use variable selection methods, such AIC, to identify the best predictors. However, such ‘causal salad’ approaches have the goal of identifying the most predictive model, and do not necessarily identify the most supported causal model (Arif and MacNeil 2022). Building a causal model could be used to identify potential confounding effects (e.g., distance from city centre) and understand the direct and indirect effects of variables (e.g., direct effects of mowing and indirect effects of mowing via changes in flowering plant richness), which will help clarify the leverage points for change in the urban system. Causal frameworks, such as structural equation models, have been applied to understand the role of different pressures within urban areas (Sanetra et al. 2024) but rarely the factors that are positively associated with urban biodiversity. In one example, Matteson et al. (2013) used structural equations to analyze the direct and indirect links between urban development, household income, floral resources and bees within urban areas. We suggest that future research could adopt more robust causal thinking to test the pathways through which green-blue infrastructure can support insect diversity. The use of a causal framework could also help understand the roles of indirect drivers, such as governance, land ownership and socio-economic variables and how they link with direct drivers associated with land management practices and vegetation cover.

Finally, despite the recognition that the design of green-blue infrastructure needs to be multidisciplinary (Parris et al. 2018), few studies have considered how insect diversity can be supported at the same time as other benefits. Future studies could address this in different ways. In one approach, a study could simultaneously measure multiple biodiversity, environmental and human outcomes at the same set of sampling sites and explore the relationships between the outcomes; for instance, to ask whether biodiversity outcomes increase with other environmental outcomes. In another approach, more process-based, the local and landscape factors affecting biodiversity, environmental and human outcomes could be gathered from different studies, which could be then used to understand which factors positively affect some outcomes but negatively affect others (leading to trade-offs) and which factors have consistent positive or negative effects across different outcomes (leading to synergies). Together, these research approaches could provide the much-needed evidence base for how to design green-blue infrastructure for people and for nature.

## Conclusions

Supporting biodiversity in urban areas is important for a range of ecosystem services, including supporting human wellbeing, as well as to engage people in conservation. Insects, however, are often overlooked in considerations about urban biodiversity despite many potential benefits. Our review provides a comprehensive synthesis - of just over 200 previous studies - that can guide insect-friendly urban planning, highlighting both the opportunities and gaps in current approaches. We find that diverse green-blue infrastructures can support insect biodiversity, but their effectiveness varies significantly based on the type of infrastructure and its management - illustrating the important role of urban planning and policy. The evidence-base could be, however, strengthened if new projects take care to collect base-line data for better attribution and quantification of the effects of new green-blue infrastructures or management practices. Our findings highlight the need for spatially and structurally diverse urban planning to accommodate different insect communities. This involves creating heterogeneous environments with diverse microclimates and habitats that can support a broad range of insect taxa. By synthesizing findings across numerous studies, we hope to provide urban planners and policymakers with actionable insights that can help prioritize interventions and design urban green-blue infrastructure that are more biodiverse and multi-functional.

## Electronic supplementary material

Below is the link to the electronic supplementary material.

Supplementary Material 1

## Data Availability

Data is provided within the associated Shiny App available at https://diana-bowler.shinyapps.io/Urban_greening_for_insects/.
