# Supplementary material for: Evidence-base for urban green-blue infrastructure to support insect diversity
Source: Urban Ecosyst. 2024 Dec 7;28(1):54. doi: 10.1007/s11252-024-01649-4 (PMC11625076; doi:10.1007/s11252-024-01649-4)
Supplement: Supplementary file 1 — Supplementary Material 1 [file 11252_2024_1649_MOESM1_ESM.docx]

#### Supplementary Information for: “Evidence-base for urban green-blue infrastructure to support insect diversity”

**Table S1**: We highlight 18 other existing relevant reviews to the topic of insect diversity in green-blue urban infrastructure.

| **Green-blue infrastructure** | **Focus** | **Reference** |
| --- | --- | --- |
| Amenity areas | Golf courses | (Colding and Folke, 2009) |
| Constructured GI | Green roofs, walls, detention basins, swales | (Filazzola et al., 2019) |
| Constructured GI | Green roofs (height and isolation) | (Blank et al., 2017) |
| Constructured GI | Green roofs | (Wang et al., 2022) |
| Gardens (flowers / pollinators) | Gardens to support pollinators | (Majewska and Altizer, 2020) |
| Gardens (flowers / pollinators) | Benefits of wildflower meadows | (Bretzel et al., 2016) |
| Gardens (flowers / pollinators) | Urban habitats for bees | (Ayers and Rehan, 2021) |
| Gardens (flowers / pollinators) | Urban bee assemblages | (Prendergast et al., 2022) |
| Waterbodies | Success of river restoration | (Al-Zankana et al., 2021) |
| Waterbodies | Permanent versus temporary bodies | (Anton-Pardo et al., 2019) |
| Waterbodies | Anthropogenic waterbodies | (Chester and Robson, 2013) |
| Waterbodies | Ponds | (Oertli and Parris, 2019) |
| Other non-sealed urban areas | Urban agriculture | (Clucas et al., 2018) |
| Other non-sealed urban areas | Unmanaged urban / vacant land | (Riley et al., 2018) |
| Not specific | Broad synthesis of local/landscape factors | (Beninde et al., 2015) |
| Not specific | Native vs non-native vegetation | (Berthon et al., 2021) |
| Not specific | Island biogeography theory | (Fattorini et al., 2018) |
| Not specific | Mowing regime | (Proske et al., 2022) |

References

Al-Zankana AFA, Smallwood LS, Matheson T, Harper DM. 2021. Hydromorphological rehabilitation improves channel morphology, instream biotopes, and macroinvertebrate communities, and thus enhances the conservation of an urban river. AQUATIC CONSERVATION-MARINE AND FRESHWATER ECOSYSTEMS 31: 2697–2713.

Anton-Pardo M, Ortega JCG, Melo AS, Bini LM. 2019. Global meta-analysis reveals that invertebrate diversity is higher in permanent than in temporary lentic water bodies. FRESHWATER BIOLOGY 64: 2234–2246.

Ayers AC, Rehan SM. 2021. Supporting Bees in Cities: How Bees Are Influenced by Local and Landscape Features. INSECTS 12.

Beninde J, Veith M, Hochkirch A. 2015. Biodiversity in cities needs space: a meta-analysis of factors determining intra-urban biodiversity variation. Ecology Letters 18: 581-592.

Berthon K, Thomas F, Bekessy S. 2021. The role of 'nativeness' in urban greening to support animal biodiversity. Landscape and Urban Planning 205.

Blank L, Vasl A, Schindler BY, Kadas GJ, Blaustein L. 2017. Horizontal and vertical island biogeography of arthropods on green roofs: a review. Urban Ecosystems 20: 911-917.

Bretzel F, Vannucchi F, Romano D, Malorgio F, Benvenuti S, Pezzarossa B. 2016. Wildflowers: From conserving biodiversity to urban greening A review. Urban Forestry & Urban Greening 20: 428-436.

Chester ET, Robson BJ. 2013. Anthropogenic refuges for freshwater biodiversity: Their ecological characteristics and management. Biological Conservation 166: 64-75.

Clucas B, Parker ID, Feldpausch-Parker AM. 2018. A systematic review of the relationship between urban agriculture and biodiversity. URBAN ECOSYSTEMS 21: 635-643.

Colding J, Folke C. 2009. The Role of Golf Courses in Biodiversity Conservation and Ecosystem Management. ECOSYSTEMS 12: 191–206.

Fattorini S, Mantoni C, De Simoni L, Galassi DMP. 2018. Island biogeography of insect conservation in urban green spaces. Environmental Conservation 45: 1–10.

Filazzola A, Shrestha N, MacIvor JS. 2019. The contribution of constructed green infrastructure to urban biodiversity: A synthesis and meta-analysis. Journal of Applied Ecology 56: 2131-2143.

Majewska AA, Altizer S. 2020. Planting gardens to support insect pollinators. Conservation Biology 34: 15-25.

Oertli B, Parris KM. 2019. Review: Toward management of urban ponds for freshwater biodiversity. Ecosphere 10.

Prendergast KS, Dixon KW, Bateman PW. 2022. A global review of determinants of native bee assemblages in urbanised landscapes. Insect Conservation and Diversity 15: 385-405.

Proske A, Lokatis S, Rolff J. 2022. Impact of mowing frequency on arthropod abundance and diversity in urban habitats: A meta-analysis. Urban Forestry & Urban Greening 76.

Riley CB, Perry KI, Ard K, Gardiner MM. 2018. Asset or Liability? Ecological and Sociological Tradeoffs of Urban Spontaneous Vegetation on Vacant Land in Shrinking Cities. Sustainability 10: 2139.

Wang L, Wang H, Wang Y, Che Y, Ge Z, Mao L. 2022. The relationship between green roofs and urban biodiversity: a systematic review. Biodiversity and Conservation 31: 1771-1796.
